# Heterosynaptic plasticity in biomembrane memristors controlled by pH

**DOI:** 10.1557/s43577-022-00344-z

**Published:** 2022-08-29

**Authors:** William T. McClintic, Haden L. Scott, Nick Moore, Mustafa Farahat, Mikayla Maxwell, Catherine D. Schuman, Dima Bolmatov, Francisco N. Barrera, John Katsaras, C. Patrick Collier

**Affiliations:** 1 Bredesen Center for Interdisciplinary Research, The University of Tennessee, Knoxville, USA; 2 Large Scale Structures Group, Neutron Scattering Division, Oak Ridge National Laboratory, Oak Ridge, USA; 3 Department of Biochemistry & Cellular and Molecular Biology, The University of Tennessee, Knoxville, USA; 4 Department of Chemical and Biomolecular Engineering, The University of Tennessee, Knoxville, USA; 5 Computer Science and Mathematics Division, Oak Ridge National Laboratory, Oak Ridge, USA; 6 Shull Wollan Center, Oak Ridge National Laboratory, Oak Ridge, USA; 7 Center for Nanophase Materials Sciences, Oak Ridge National Laboratory, Oak Ridge, USA

**Keywords:** Membrane, Neuromorphic, Biomimetic, Synaptic plasticity, Interface

## Abstract

**Abstract:**

In biology, heterosynaptic plasticity maintains homeostasis in synaptic inputs during associative learning and memory, and initiates long-term changes in synaptic strengths that nonspecifically modulate different synapse types. In bioinspired neuromorphic circuits, heterosynaptic plasticity may be used to extend the functionality of two-terminal, biomimetic memristors. In this article, we explore how changes in the pH of droplet interface bilayer aqueous solutions modulate the memristive responses of a lipid bilayer membrane in the pH range 4.97–7.40. Surprisingly, we did not find conclusive evidence for pH-dependent shifts in the voltage thresholds (V*) needed for alamethicin ion channel formation in the membrane. However, we did observe a clear modulation in the dynamics of pore formation with pH in time-dependent, pulsed voltage experiments. Moreover, at the same voltage, lowering the pH resulted in higher steady-state currents because of increased numbers of conductive peptide ion channels in the membrane. This was due to increased partitioning of alamethicin monomers into the membrane at pH 4.97, which is below the pKa (~5.3–5.7) of carboxylate groups on the glutamate residues of the peptide, making the monomers more hydrophobic. Neutralization of the negative charges on these residues, under acidic conditions, increased the concentration of peptide monomers in the membrane, shifting the equilibrium concentrations of peptide aggregate assemblies in the membrane to favor greater numbers of larger, increasingly more conductive pores. It also increased the relaxation time constants for pore formation and decay, and enhanced short-term facilitation and depression of the switching characteristics of the device. Modulating these thresholds globally and independently of alamethicin concentration and applied voltage will enable the assembly of neuromorphic computational circuitry with enhanced functionality.

**Impact statement:**

We describe how to use pH as a modulatory “interneuron” that changes the voltage-dependent memristance of alamethicin ion channels in lipid bilayers by changing the structure and dynamical properties of the bilayer. Having the ability to independently control the threshold levels for pore conduction from voltage or ion channel concentration enables additional levels of programmability in a neuromorphic system. In this article, we note that barriers to conduction from membrane-bound ion channels can be lowered by reducing solution pH, resulting in higher currents, and enhanced short-term learning behavior in the form of paired-pulse facilitation. Tuning threshold values with environmental variables, such as pH, provide additional training and learning algorithms that can be used to elicit complex functionality within spiking neural networks.

**Graphical abstract:**

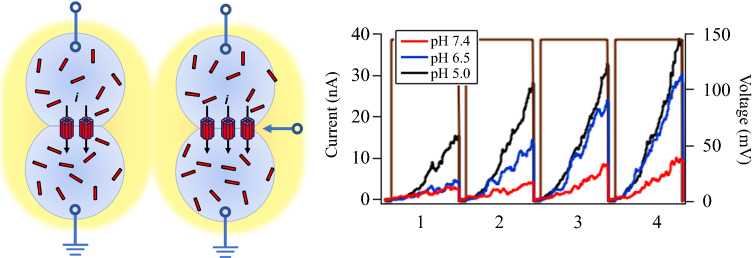

**Supplementary information:**

The online version contains supplementary material available at 10.1557/s43577-022-00344-z.

## Introduction

Synaptic plasticity refers to the ability of a synaptic connection between neurons to change its strength. Homosynaptic plasticity refers to synaptic connections that are input specific, meaning that activity at a specific neuron is responsible for the strength of the synaptic connections with that neuron. However, there are also examples of heterosynaptic plasticity, or synaptic plasticity, involving much larger populations of synapses and neurons, where specific synaptic connections are not directly targeted. In biology, heterosynaptic plasticity maintains homeostasis in synaptic inputs during associative learning and memory and can initiate extended, long-lasting changes in synaptic strengths that are not specific to any one synapse, but can indirectly modulate many synapses in an extended neural circuit.^1^

Heterosynaptic plasticity may extend the functionality of bioinspired neuromorphic circuits consisting of memristors and memcapacitors by enabling additional parameters with which to modulate short-term and long-term synaptic plasticity in these circuits. It involves “interneurons” that can modulate the communication efficiency of synapses in neural circuits without affecting any one synapse.^2^ Interneurons in solid-state devices can be gate electrodes in three-terminal synaptic transistors,^3^ or auxiliary structures that can apply electric and magnetic fields that modulate memristive properties locally between the presynaptic and postsynaptic electrodes in tunnel junctions.^4^ In two-terminal soft-matter memristors based on lipid bilayer membranes and membrane-associated ion channels, interneurons could be developed to globally modulate the voltage-dependent conductance and capacitance of the bilayer. These would not necessarily require a physical third electrode, but instead, could be based on environmental changes affecting many synapses, such as pH, ionic strength, and temperature. These external variables can change the structure and dynamical properties of ion channels and of the bilayer in which they reside.

Changes in pH can also be used to titrate the ionizable groups of charged lipid bilayers, such as phosphatidylserine (PS), which has three ionizable groups and is negatively charged at neutral pH. Phosphatidylcholine (PC), a frequently used component of lamellar lipid bilayers, is zwitterionic, and therefore, net neutral without ionizable groups, except at extreme values of pH. Nevertheless, even relatively “modest” changes in pH can charge PC lipid bilayers with embedded ion channels, similarly to those in charged PS lipid bilayers.^5^

Previously, we reported on short-term synaptic plasticity in artificial synapses via the memristive behavior in alamethicin-doped diphytanoylphosphatidylcholine (DPhPC) lipid membranes. DPhPC is a synthetic PC lipid known for its chemical stability and low ion permeability in droplet interface bilayers (DIBs), a membrane platform that consists of two aqueous droplets in oil (hexadecane), each coated with a monolayer of lipids that form a lipid bilayer between them.^6,7^ For this study, we explored how changes in pH modulate DIB memristive responses. A reasonable expectation is that the largest observable effect would be a shift in the voltage threshold (V*) needed to form conductive pores in the membrane. These thresholds are important components of memristive behavior in that they are partially responsible for the “pinched hysteresis” in current–voltage (*I*/*V*) plots, a hallmark of a memristive system.^8^ Surprisingly, we were not able to definitively link changes in pH with shifts in V* beyond random noise. This does not necessarily mean that such a link does not exist. Even at the same pH, the V* values we measured were stochastic, and any link with changes in pH may have been so weak that they were overwhelmed by the system’s intrinsic noise. We did, however, find a clear link between pH and the time-dependent current responses to voltage pulses, which can also be used to detect and categorize memristive behavior. At low pH, we found increased current levels at the same V*, pore conduction onset at lower V* values, and enhanced short-term synaptic plasticity in the form of increased paired-pulse facilitation (PPF) of the switching conductance. We anticipate that these findings will help in constructing neuromorphic circuitry with enhanced functionality.

## Results

**Figure 1:**
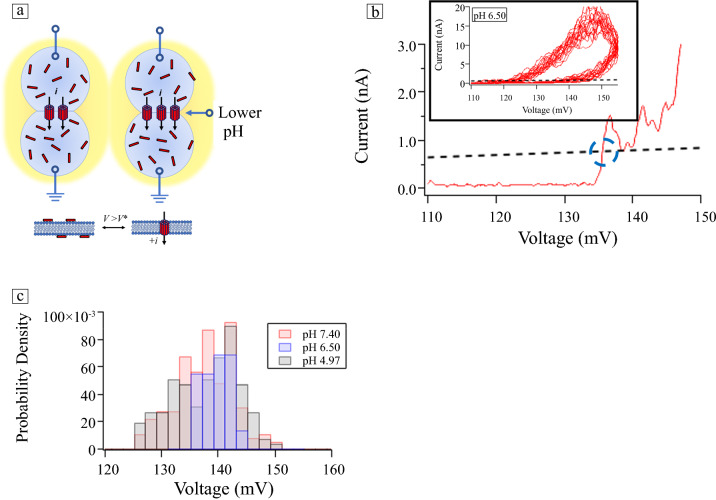
Process flow for characterizing shifts in V* with changes in pH of droplet interface bilayers (DIBs) doped with alamethicin. (a) Schematic for *V* > V* showing alamethicin undergoing a transition from monomers lying parallel to the bilayer surface (S-state), to their fully inserted state (I-state), where they oligomerize into pores. (b) Inset: Evolution of the positive lobe of sequential pinched hysteresis loops generated through cyclic voltammetry of alamethicin—DPhPC DIBs. Main: The first *I*/*V* pass through the first hysteresis loop crosses the minimum detectable current threshold (black-dashed line, 8 μS/cm^2^) at V* (highlighted with blue-dashed circle). (c) Histograms of numerous V* values at each of the three pH values studied, fitted to Gaussian distributions. The differences in the mean values for V* were less (1–2 mV) than the widths (variances) of the distributions (4–9 mV), suggesting that any shifts in V* were, most likely, much smaller than the noise.

**Figure** **1** is an overview of the process used to detect shifts in voltage threshold, V*, values with changes in pH. In Figure 1a, above a characteristic V*, alamethicin monomers undergo a phase transition from a surface-associated (*S*) state, where the long axis of the channel forming peptide is parallel to the plane of the bilayer, to an inserted (*I*) state, where monomers in the membrane oligomerize into conductive pores. In this scenario, increases in ionic current for voltages greater than a voltage threshold (*V* > V*) are the result of a higher number of conductive pores in the membrane, rather than an increase in pore conductance.^9,10^ This voltage threshold results in the well-known “pinched hysteresis” phenomenon associated with memristive behavior in alamethicin-DPhPC bilayer membranes.^7^ This is the result of lag times between the formation of conductive pores in the membrane, governed by the voltage threshold at V*, and voltage-dependent changes to the membrane area due to electrowetting.

The inset to Figure 1b shows the growth of the positive lobe of a hysteresis loop generated by steady-state currents from alamethecin pores in DPhPC bilayers, as functions of voltage. The main graph shows the first current versus voltage trajectory of the loop, which extends from zero volts to *V* > V* threshold for ion channel conduction. This threshold is defined as 10× the background conductance, which is 8 μS/cm^2^ (plotted as the black-dashed line in the figure).^11^ V* is defined as the voltage where the current from the alamethicin-DPhPC bilayer first overtakes this threshold current (blue-dashed circle)—the majority of the threshold crossings occurred later and at lower voltages. Figure 1c shows probability density distributions as histograms of V* at three different pH values (pH 4.97, 6.50, and 7.40). The three distributions were well fitted to Gaussian functions (SI) and aligned closely enough with each other that the differences in their mean values (1–2 mV) were much smaller than the variances (widths) of their distributions (4–9 mV), indicating that noise levels were random and not correlated to changes in pH.

The number of monomers that form a channel in the lipid bilayer and the charge per monomer that crosses the membrane both affect V*. This is because alamethicin-induced conduction is dependent on both voltage and peptide concentration.^12^ The V* probability density distributions shown in Figure 1c will most likely change with changes in peptide concentration, to the extent that the V* distributions at the three different pH values may become resolvable given enough of a concentration difference. The peptide concentration was kept constant ([alamethicin] = 3 μM) at a low enough level that only voltage was responsible for ion channel formation in the membrane.^7^ For comparison, the peptide concentration threshold for alamethicin pore formation in the absence of voltage is about 20 μM.^11^

We found that a better strategy for characterizing memristive behavior as a function of peptide concentration is with the time-dependent dynamics of ionic currents in response to voltage pulses. At constant peptide concentration, the dynamic state equation for the number of open pores in the membrane, *N*_*a*_, can be expressed as a first-order kinetic equation:^7,13^

1 $$\frac{d{N}_{a}}{dt}=n-m{N}_{a},$$

where *n* represents the rate of conductive pore formation (pores/s cm^2^) and *m* is the rate of pore decay (1/s). This equation can be solved to give an expression for *N*_*a*_ as a function of time:

2 $${N}_{a}=\frac{n}{m}\left(1-{e}^{-mt}\right).$$

The ratio of the rates, ${N}_{\text{as}}=n/m$ (pores/cm^2^), is the number of open pores under steady-state conditions. Both rates are strongly dependent on voltage, given by the following relations:

3 $$n={n}_{0}{e}^{V/{V}_{n}}, m={m}_{0}{e}^{V/{V}_{m}},$$

where *n*_0_ is the pore formation rate at 0 V, which for alamethicin has been determined to be about 10^3^ pores/s cm^2^, and *m*_0_ is the corresponding pore decay rate at 0 V, roughly 20 s^−1^.^13^ *V*_*n*_ and *V*_*m*_ are the voltages required to increase the pore formation rate or pore decay rate *e*-fold. Together, they are responsible for the rapid increase in the number of open pores per unit area with applied voltage:

4 $${N}_{a}=\frac{{n}_{0}}{{m}_{0}}{e}^{V\left(\frac{1}{{V}_{n}}+\frac{1}{{V}_{m}}\right)}.$$

**Figure 2:**
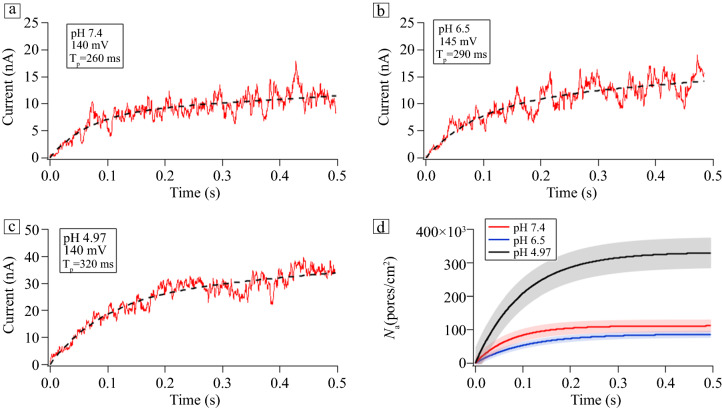
Changes in ionic currents from open alamethicin pores in response to 500-ms-long voltage pulses separated by 2000 ms, as functions of voltage and changes in pH: (a) pH 7.40, (b) pH 6.50, (c) pH 4.97. The stimulating voltage for both pH 7.4 and pH 4.97 was 140 mV, and for pH 6.5, 145 mV. The black-dashed lines are fits of the currents to Equation 9. Text insets contain the potentiation time constants, *τ*_p_, defined as the times for the number of conductive pores in the membrane to reach steady-state values. (d) The number of conductive pores per unit area (Equation 2) for the three different pH-applied voltage combinations studied, which were determined from the fitted currents. The shaded region around each trace corresponds to one standard deviation from the mean.

**Figure** **2** shows how pH modulates memristance in the membrane in response to square voltage pulses, each of 0.5 s in duration and separated by 2.0 s. For Figure 2a, c, at pH 7.40 and pH 4.97, respectively, the exciting voltage was 140 mV, whereas for Figure 2b, at pH 6.50, it was 145 mV. This provided insight into the relative importance of pH versus voltage, as well as helping to identify possible interactions between the two. Figure 2a–c shows a representative current trace and the mean potentiation time constant, *τ*_p_, averaged over seven independent trials. These values correspond to the times needed for the number of open pores in the membrane, *N*_*a*_, to reach steady-state values.

Each current trace in Figure 2a–c was fitted to an equation for the current (shown as black-dashed lines aligned with the current traces) that included *N*_*a*_, the time-dependent number of open pores per unit area in the membrane, and an additional term for electrowetting, which consisted of an increase in lipid bilayer area with voltage, driven entropically by the expulsion of oil between the droplets as a function of applied voltage.^14^ The voltage and time dependence of the currents are given by^7^

5 $$i\left(V,t\right)={G}_{u}{N}_{a}\left(V,t\right)A\left(V,t\right)V,$$

where *G*_*u*_ is the ensemble-averaged conductance of a single alamethicin pore, for example, 1.03 nS,^14^ *N*_*a*_ (*V*, *t*) is the voltage- and time-dependent number of open pores in the membrane per unit area, and *A* (*V*, *t*) is the bilayer area, also dependent on both voltage and time. This expression can be resolved into contributions from *N*_*a*_, given by Equation 2, and the membrane area, given by^7^

6 $$A\left(V,t\right)={A}_{0}\left(1+{A}_{m}\left(V,t\right)\right),$$

where *A*_0_ is the membrane area at zero volts and *A*_*m*_ is the fractional increase in membrane area at an applied voltage:

7 $${A}_{m}\left(V,t\right)=\frac{A\left(V,t\right)-{A}_{0}}{{A}_{0}}.$$

The dynamical response of *A*_*m*_ is given by

8 $$\frac{d{A}_{m}(V,t)}{dt}=\frac{1}{{\tau }_{ew}}\left(\alpha {V}^{2}-{A}_{m}(V,t)\right),$$

where *τ*_*ew*_ is a characteristic time constant for electrowetting (about 1.5 s for alamethicin-DPhPC bilayers at room temperature) and *α* is a gain coefficient applied to fractional increases in membrane area such that, at steady state, *A*_ms_ = α*V*^2^.^7^

The form for the fitted equation for current (Equation 5) then becomes

9 $$i\left(V,t\right)={G}_{u}\left[\frac{n}{m}\left(1-{e}^{-mt}\right)\right]\left[1+bt\right]{A}_{0}V.$$

On inspection, the first bracketed term in Equation 9 is Equation 2, whereas the second term is given by Equation 6. These terms range from 0 up to their voltage-dependent, steady-state values, given by *N*_as_ = *n*/*m* for the numbers of open pores per unit area, and *bt* = *A*_ms_ = α*V*^2^ for the fractional increases in membrane areas.

Fitting the current responses to Equation 9 enables one to remove the bulk-scale, voltage-dependent bilayer electrowetting terms from the inherent voltage-dependent pore formation, and membrane decay dynamics takes place at the nanoscale. The results for the three pH values are depicted in Figure 2d, which shows the number of conductive pores per unit area for the three different pH/applied voltage combinations studied, determined from the fitted currents of seven individual trials conducted at each pH. The “potentiation” times for reaching the steady-state numbers of open pores in the membrane, *τ*_p_, were determined graphically from Equation 9 after removing the electrowetting term (SI). They are annotated in the text box for each of the three pH values shown in Figure 2a–c. The fitted values for the mean and standard deviation of *n*, *m*, and *n*/*m* at each pH are listed in **Table** **I**, and one standard deviation from the mean is plotted in Figure 2d (shown as the shaded region around each trace at 68% confidence interval).

**Table I:** Fitted parameters for the pulsed currents (Equation 9, Figure 2) at the three pH-bias voltage combinations, averaged over seven independent trials.

	Pore Formation Rate (pores/s cm^2^) (*n*)	Pore Decay Rate (1/s) (m)	Steady-State *N*_as_ (pores/cm^2^) (*n*/m)	*τ*_p_ (ms)	Fractional Area Increase, *A*_m_/*t* (1/s) (*b*)
pH 7.40, 140 mV *A*_0_ = 5.9 × 10^−4^ cm^2^	1.5 × 10^6^ ± 4.5 × 10^5^	13.8 ± 5.2	1.1 × 10^5^ ± 1.2 × 10^4^	260	0.72
pH 6.50, 145 mV *A*_0_ = 8.6 × 10^−4^ cm^2^	8.1 × 10^5^ ± 2.1 × 10^5^	9.4 ± 2.7	8.6 × 10^4^ ± 3.6 × 10^3^	290	0.72
pH 4.97, 140 mV *A*_0_ = 5.7 × 10^−4^ cm^2^	3.2 × 10^6^ ± 4.1 × 10^5^	9.9 ± 1.7	3.3 × 10^5^ ± 1.9 × 10^4^	320	0.64

They include alamethicin pore formation and decay rates, steady-state numbers of channels, and potentiation times to reach steady-state current levels.

The most noticeable difference occurs at pH 4.97 (Figure 2c–d), which has a significantly larger steady-state number of open pores (*N*_as_) than either pH 6.5 or pH 7.4. These two traces are close to each other—and almost overlap—but the steady-state number of conductive pores at pH 6.5 are clearly lower than those at pH 7.4, even though the applied voltage at pH 6.5 was 5 mV higher (applied voltage 145 mV for pH 6.5 versus 140 mV for pH 7.4 and pH 5.0).

**Figure 3:**
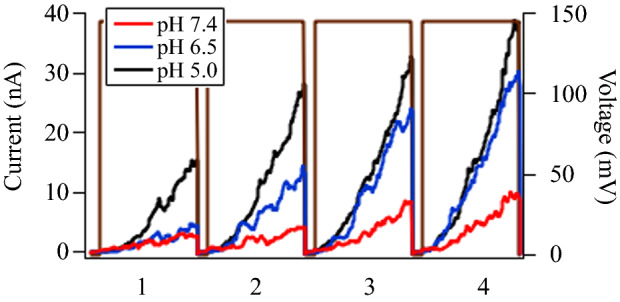
Changes in paired-pulse facilitation from alamethicin-doped DPhPC memristors with changes in pH, to a series of 145 mV, 50 ms pulses separated by 10 ms off-times, at pH 5.0 (black trace), pH 6.5 (blue trace), and pH 7.4 (red trace).

**Figure** **3** depicts how changes in pH modify paired-pulse facilitation (PPF), which is a form of short-term synaptic plasticity (STP) triggered in the presynaptic neuron. PPF and its opposite, paired-pulse depression (PPD), manifest as two presynaptic spikes evoked in succession.^7^ Figure 3 shows the response of an alamethicin-doped DPhPC memristor to a series of 145 mV, 50 ms pulses separated by 10 ms off-times at the three pH values studied, all at room temperature. All three pH values show successive increases in current with each pulse, typical of PPF behavior. However, there are also striking pH dependencies that can be observed at the individual pulse level (i.e., compare the three currents as functions of pH separately for each pulse in Figure 3). It appears from Figure 3 that the current at pH 6.5 had the largest relative increase (comparing pulse 1 with pulse 4). This trend was repeated over multiple trials, although the individual current amplitudes were stochastic. Figure S3 shows the extended, time-dependent scans of current pulses at the three pH values from which the pulse sequences in Figure 3 were taken.

It is important to highlight the fact that the currents, and hence PPF, also had a very strong, nonlinear voltage dependence (related to V*), which must be accounted for in addition to pH. At lower bias voltages (130 mV and below), more acidic conditions were required not only for pore formation and ionic conduction, but to obtain unambiguous signatures of PPF and PPD (Figure S4). In other words, an environment with moderate acidity increases the likelihood of a lipid bilayer membrane with embedded alamethicin ion channels exhibiting STP. This is consistent with increased relaxation times at lower pH, which are the reciprocals of the pore decay rates, *τ*_r_ = 1/*m*.

## Discussion

Observations from Table I and Figure 2 that help deduce the connections between pH, voltage, and memristive behavior include

1. The pore formation rate (*n*) and steady-state number of pores in the membrane (*n*/*m*) at pH 4.97 are significantly larger than at pH 6.5 or 7.40.
2. The pore formation rate (*n*) at pH 6.5 is almost a factor of two less than at pH 7.4.
3. The pore decay rate (*m*) at pH 7.4 is larger than at pH 4.97 and pH 6.5, which are similar.
4. The electrowetting contributions to the pulsed currents (*b*) are higher at pH 6.5 and pH 7.4 than at pH 4.97.
5. The potentiation times to reach steady-state behavior are similar for the three pH values studied.
6. Short-term synaptic plasticity behaviors such as PPF are amplified at lower pH.

A reasonable starting point for a realistic membrane model with properties consistent with these observations is to combine the individual contributions from (a) alamethicin pore formation and decay rates; and (b) time-dependent electrowetting of the bilayers. For (a), early studies characterizing the pore formation and decay dynamics of antimicrobial peptides (AMPs) such as alamethicin in lipid bilayers have been primarily carried out with black lipid membranes (BLMs), which are single lipid bilayers “painted” across a hydrophobic aperture separating two aqueous compartments. Unlike DIBs, BLMs lack an oil phase and hence, do not have an electrowetting term similar to that in Equation 9.^12^ In a BLM, the time course of the current response to voltage pulses has only two contributions. At early times, the first contribution is given by $n/m\left(1-{e}^{-mt}\right)$, which describes the time-dependent growth in the number of conductive open pores up to a constant, steady-state level defined by $n/m$, for example, the balanced ratio of pore formation to pore decay rates. For case (b), because of electrowetting, the steady-state regime in DIBs is no longer a constant value but increases linearly with time under the influence of the second bracketed term in Equation 9, namely the change in the fractional membrane area. The time dependence for electrowetting is given by Equation 6.

The values of the fitted parameters in Table I are consistent with models that focus on how surface charge affects pore formation in AMPs like alamethicin^15^—charge plays a major role in how biological membranes interact with molecules. For example, it has been shown that changes in the surface potential and surface charge density by proton titration of the ionizable groups in charged PS lipids result in changes to the elastic properties of the bilayer.^15,16^ In the case of alamethicin-DPhPC bilayers, two titrations of exchangeable hydrogens can occur as a function of pH, namely, titration of the membrane surface charge, and titration of the alamethicin channel conductance.

Titration of the bilayer surface charge strongly affects the hydrophobic matching of the peptide pore with the bilayer, a critical parameter for peptide insertion, and pore formation in the membrane. Hydrophobic mismatches resulting from charge cause the system to move away from conformational equilibrium by inducing high lateral pressures on alamethicin, due to headgroup electrostatic repulsion and reduced conformational entropy within the hydrophobic core of the membrane.^17^ For a well-ordered bilayer, this pressure is usually balanced by a negative lateral tension at the aqueous interface. However, this balance can be easily disrupted by the nucleation of defects resulting from the distortion of the membrane in the vicinity of the ion channel. Zwitterionic lipids, such as DPhPC, are also affected by added charge but in different ways than charged lipids. At pH 7.40, there are more OH^−^ ions than H^+^ ions in solution. Also, because OH^−^ ions are polarizable and H^+^ ions are not, we expect OH^−^ to associate more with the PC headgroups, resulting in a net negatively charged membrane^18^ that persists until the isoelectric point of PC lipids is reached (determined from electrophoretic mobility measurements of vesicles), which occurs near pH 4.

In terms of the acid-titratable groups on DPhPC, the pKa of both the phosphate (pKa ~ 1) and choline (pKa ~ 11) groups are clearly out of bounds. However, the pKa of alamethicin’s negatively charged carboxylate groups on glutamate residues that line the lumen of the pore is reported to be pKa ~ 5.3–5.7, not unlike those of other antimicrobial membrane peptides.^9^ This is well within the range of pH values studied here (i.e., pH 7.4, pH 6.5, and pH 4.97).^16^ At pH 4.97, these charged groups are neutralized, stabilizing pore formation and the number of open pores in the bilayer by screening the electrostatic repulsion of α-helix dipoles of the peptide.^19^ The other two pH values are both above the pKa value of the peptide but were maintained at different voltages from each other (the pulses at pH 6.50 were kept at 145 mV, whereas the other two pH values were pulsed at 140 mV). In general, increasing the stimulating voltage from 140 to 145 mV will increase both the pore formation and decay rates, as shown in Equation 3, and the membrane area via electrowetting, which has a quadratic dependence with voltage (Equation 8). From our experiments, however, the pH change from 7.4 to 6.5 seems to have a larger influence than the change in bias voltage.

The accepted model for pore formation for alamethicin involves a rotation and oligomerization of peptide monomers in the membrane.^12^ Alamethicin’s large dipole moment (70–80 D) plays an important role in its voltage-dependent gating mechanism. Upon application of a voltage greater than V*, the interaction of the dipole with the electric field causes alamethicin monomers to rotate and insert into the membrane, aligning with the lipid fatty acid tails. Once inserted, peptide monomers can diffuse within the membrane, eventually forming oligomeric conductive pores. The peptide’s negatively charged glutamate residues at neutral pH near its C-terminus are thought to help anchor it in the membrane through a network of hydrogen bonds with water and the lipid headgroups, allowing the positively charged N-terminus to traverse the membrane during insertion. However, lowering the pH causes OH^−^ ions to partition into the membrane, thus, changing both the surface charge and the electric field experienced by alamethicin pores, which affects the mechanism for peptide insertion in the membrane and oligomerization leading to pore formation,^19,20^ as evidenced in Figure 2d by the slightly reduced steady-state number of pores per unit area at pH 6.5 versus 7.4.

The sharp increase in pore activity that we observed at pH 4.97 on the other hand is consistent with reports of enhanced probability for alamethicin pore formation in more acidic environments.^15^ Although increased acidity enhanced the probability of a pore being in a higher conductance state, the channel conductance itself was largely unaffected.^15^ This is an important observation because it shows that the enhanced currents at lower pH are due mainly to increases in the number of active channels in the membrane, and not intrinsic pore conductance. For alamethicin, aggregation of peptide monomers to form pores reduces energetically costly peptide–lipid interactions due to hydrophobic mismatch. In this case, transitions between different conductance states correspond to the reversible addition of monomers to an existing pore as a function of voltage. As the concentration of peptide monomers increases, positive cooperativity for attraction to the lipids in the membrane emerges due to oligomerization. Aggregates cannot dissociate from the membrane as easily as monomers can provide a thermodynamic driving force for pore formation.^13^ Higher conductance states correspond to larger oligomers. This results in a distribution of pore sizes in the membrane that corresponds to the distribution of conductance levels.^21^

Partitioning of peptide monomers from the aqueous phase to the membrane is largely driven by the hydrophobicity of the peptide. The overall hydrophobicity is related to the peptide’s amino acid composition, and it is well known that alamethicin’s 20 different amino acids have different partitioning behaviors between polar and nonpolar environments.^22^ Additionally, the naturally charged amino acids have variable hydrophobicity values that are dependent on their charge state. Looking specifically at glutamate, the free energy of transfer, ΔG, of this residue from water to a POPC bilayer decreases from 2.02 kcal/mol in its negatively charged state to −0.01 kcal/mol when protonated, leading to a favorable transfer of peptide from water to the surface of a POPC bilayer.^22^ Here, we observe that pore formation rates, pore decay rates, and the steady-state numbers of active channels all increase as a function of decreasing pH below the pKa of the two glutamate residues in alamethicin (pKa ~ 5.3–5.7). This can be rationalized by an increase in alamethicin hydrophobicity, and consequently, increased alamethicin monomer partitioning to the membrane from the aqueous phase. With more alamethicin monomers on the membrane surface at pH 4.97 compared to 7.4, passing the voltage threshold for insertion leads to a greater number of pores that are formed faster and are more stable. This is similar to the membrane association and insertion process of the pH-low insertion peptide (pHLIP), where changes in peptide hydrophobicity increase due to protonation of charged amino acid residues, culminating in membrane insertion.^23–25^

## Conclusions

We found that decreasing pH below the pKa of the glutamate residues of alamethicin resulted in increased ionic current levels, due to increased partitioning of alamethicin monomers in the membrane, and stabilization of the pore lumen of the oligomers by screening the electrostatic repulsion of α-helix dipoles of the peptide. Lowering pH also resulted in the neutralization of the initially negatively charged DPhPC bilayer due to hydroxide anions that had nonspecifically migrated in the bilayer at pH 7.4. This amplified the hydrophobic mismatch between lipids and peptides by locally increasing membrane curvature stress at the pore.^20,26^ These considerations are captured by the elementary steps involved in the derivation of the state equations describing the memristive behavior of alamethicin-doped DPhPC lipid bilayers.^7^

This is also an example of how collective motions in the membrane impart “force from lipid” effects on biomembrane molecules.^26^ These local forces can modulate mechanisms of protein function and are seen most often in mechanosensitive ion channels but have been observed in many other contexts, including alamethicin pore formation described here. Changing pH changes the ionic currents from alamethicin pores in a similar manner as changing the voltage,^27^ except for the fact that here it is an indirect effect that can extend over many synapses and neurons within the context of heterosynaptic plasticity. These changes also enhance short-term facilitation and depression of the switching characteristics of the device. Figures 3, S3, and S4 show changes in STP, which repeat the experiments by Najem et al.^7^ at three different pH values. Both paired-pulse facilitation and paired-pulse depression, two canonical examples of STP, were enhanced under acidic conditions, consistent with the observed increases in the pore relaxation times (*τ*_r_ = 1/*m*, from Table I).

Recently, we reported a downward shift in the V* threshold voltages for alamethicin pore formation as a function of aqueous macromolecular crowding in a DIB system, but in that case, it was due to an increased chemical potential of alamethicin monomers at the membrane and increased osmotic stress in the bilayers due to excluded volumes in the pores that were inaccessible to the water-soluble, high-molecular-weight polymers used as crowders.^28^ Those changes resulted in a large enough effective concentration increase in peptide monomers due to attractive entropic depletion effects at the membrane to change V*, which was not the case here. Instead, the distributions of V* values do not seem to change with changes in pH beyond random error. Moreover, the chemical potentials of the peptide monomers at the alamethicin concentrations used here (3 μM) were not high enough to result in clear changes in V*, which depends on concentration. However, the kinetics for pore formation in pulsed experiments did change with pH in predictable ways that could be rationalized.

Thresholds are ubiquitous in neuromorphic networks, starting with the earliest networks known as perceptrons, which featured thresholds as the defining computational element.^29^ Many biological systems use time differences between action potentials (“spikes”) to encode information, which has led to the development of artificial networks of “spiking neurons” as computational elements. Also known as “integrate-and-fire neurons,” these model systems rely on thresholds in both voltage, current, and time (i.e., refractory periods) for computation. Soft-matter neuromorphic devices like the biomolecular memristors described here can be configured as spiking neural networks (SNNs), which are ideally suited for processing temporal data at a fraction of the energetic cost and number of resources (synapses, neurons) needed in traditional convolution-based neuromorphic networks.^30^ Heterosynaptic plasticity in bioinspired SNNs, enabled by tuning environmental variables such as pH, can provide additional programmable elements that will make these models more biologically realistic, and enhance both their functionality and flexibility for future AI and machine learning applications.

## Materials and methods

### Materials

Potassium chloride (KCl), 3-(*N*-morpholino)propanesulfonic acid (MOPS) buffer, sodium acetate (NaOAc), sodium hydroxide, agarose powder (p/n A9539), and ethanol were obtained from Sigma-Aldrich. Glacial acetic acid was purchased from EMDMillipore. Alamethicin was dissolved in ethanol to a concentration of 5 mg/mL to create a stock solution used for further sample preparation, and the stock solution was stored at −20°C when not in use. Liposome solutions were prepared by dissolving 1,2-diphytanoyl-*sn*-glycero-3-phosphocholine (DPhPC) lipids (Avanti, Alabaster, AL) at 2 mg/mL in buffer (10 mM MOPS or 100 mM NaOAc and 500 mM KCl. To obtain pH 7.45 or 6.5 with MOPS, NaOH was used to adjust the pH of MOPS dissolved in H_2_O. NaOAc was prepared at pH 5 by mixing sodium acetate with glacial acetic acid in a 3:1 ratio. The resulting multilamellar vesicles were extruded 31 times through a mini-extruder (Avanti) containing a track-etched 100-nm polycarbonate membrane creating large unilamellar vesicles (LUVs).

### Assembly

Synaptic mimic assembly was based on the droplet interface bilayer (DIB) method, which has been used extensively in recent years to study the biophysics of bioarchitectural memristive systems.^7^ Concentrations for peptides were assigned using the molar ratio of available lipid to peptide and were L/P = 788 (1 µM alamethicin). Peptides were suspended in aqueous buffer at 500 mM KCl and 2.4 mM DPhPC (as 100 nm extruded unilamellar vesicles) unless mentioned otherwise. Aqueous droplets of 300 nL volume were manually pipetted to agarose-coated silver/silver-chloride electrodes. Data were recorded using a patch clamp amplifier (Axopatch 1D, Molecular Devices, San Jose, CA). Capacitive current response to 10 Hz, 10 mV triangular voltage sweeps (Agilent) was used to monitor bilayer formation and thickness.^14^ Bright-field images were acquired using the 4× objective of an inverted Nikon TE-300 optical microscope. Recording and analysis alamethicin activity was assessed in response to a cyclic triangular voltage waveform, using bipolar cyclic voltammetry (CV) scans. Scan rates were run at 100, 250, and 500 mV/s (Stanford Research Systems D345). Amplitudes were chosen to elicit current responses greater than 1 nA. Alamethicin was added in equal amounts to both sides of the membrane. Aqueous buffer and electrolyte were also identically added to each side of the DIB. Quantitatively, conductive pore formation was achieved once the specific membrane conductance in the presence of alamethicin increased beyond a threshold of G^*^ = 8 μS/cm^2^ (Figure 1b), which we estimate to be about an order of magnitude greater than the background conductance of a DPhPC lipid bilayer without the presence of alamethicin. The voltage that gives rise to the specific current that crosses the 8 μS/cm^2^ line is the threshold voltage, V*, which must be exceeded to create a conductive pore. The conductance can then be expressed as *G* = *i*/(*V*−V^*^), which requires *V* > V^*^ for the onset of nonzero currents.^31^

Current/voltage (I/V) plots were generated from the averages of five consecutive time-dependent segments taken from bipolar CV scans of DPhPC lipid bilayers with alamethicin channels at three different scan rates, 100, 250, and 500 mV/s, after removing the capacitive currents.^32,33^ Histograms of V* values for the rising and falling segments at each scan rate were generated with a script written in Igor Pro programming language (WaveMetrics) from numerous (ca. 100) I/V curves and converted to probability density distributions by normalizing the total areas under the curves to one.

## Supplementary information

Below is the link to the electronic supplementary material.

Supplementary file1 (DOCX 3058 kb)

## Data Availability

The data sets generated and/or analyzed during the current study are available from the corresponding author on reasonable request.

## References

[1] Bailey CH, Giustetto M, Huang YY, Hawkins RD, Kandel ER (2000). Is heterosynaptic modulation essential for stabilizing hebbian plasticity and memory?. Nat. Rev. Neurosci..

[2] D. Purves, G.J. Augustine, D. Fitzpatrick, W.C Hall, A.S LaMantia, L.E. White, “Synaptic Plasticity,” in *Neuroscience*, 5th ed. (Sinauer Associates, Sunderland, MA), pp. 163–182

[3] Long TY, Zhu LQ, Ren ZY, Guo YB (2020). Global modulatory heterosynaptic mechanisms in bio-polymer electrolyte gated oxide neuron transistors. J. Phys. D Appl. Phys..

[4] Yang Y, Chen B, Lu WD (2015). Memrisitive physically evolving networks enabling emulation of heterosynaptic plasticity. Adv. Mater..

[5] Patelska AD, Figaszewski ZA (2000). Effect of pH on the interfacial tension of lipid bilayer membranes. Biophys. J..

[6] Andersson M, Jackman J, Wilson D, Jarvoll P, Alfredsson V, Okeyo G, Duran R (2011). Vesicle and bilayer formation of diphytanoylphosphatidylcholine (DPhPC) and diphytanoylphosphatidylethanolamine (Dphpe) mixtures and their bilayers’ electrical stability. Colloids Surf. B.

[7] Najem JS, Taylor GJ, Weiss RJ, Hasan MS, Rose G, Schuman CD, Belianinov A, Collier CP, Sarles SA (2018). Memristive ion channel-doped biomembranes as synaptic mimics. ACS Nano.

[8] Chua L (2014). If it’s pinched, it’s a memristor. Semicond. Sci. Technol..

[9] Zasloff M (2002). Antimicrobial peptides of multicellular organisms. Nature.

[10] Huang HW (2000). Action of antimicrobial peptides: two-state model. Biochem..

[11] Taylor GJ, Sarles SA (2015). Heating-enabled formation of droplet interface bilayers using *Escherichia coli* total lipid extract. Langmuir.

[12] Hall JE, Vodyanoy I, Balasubramanian TM, Marshall GR (1984). Alamethicin, a rich model for channel behavior. Biophys. J..

[13] Eisenberg M, Hall JE, Mead CA (1973). The nature of the voltage-dependent conductance induced by alamethicin in black lipid membranes. J. Membr. Biol..

[14] Taylor GJ, Venkatesan GA, Collier CP, Sarles SA (2015). Direct in situ measurement of specific capacitance, monolayer tension, and bilayer tension in a droplet interface bilayer. Soft Matter.

[15] Bezrukov SM, Rand RP, Vodyanoy I, Parsegian VA (1998). Lipid packing stress and polypeptide aggregation: alamethicin channel probed by proton titration of lipid charge. Faraday Discuss..

[16] Bezrukov SM (2000). Functional consequences of lipid packing stress. Curr. Opin. Colloid Interface Sci..

[17] Killian JA (1998). Hydrophobic mismatch between proteins and lipids in membranes. Biochim. Biophys. Acta.

[18] Cantor RS (1999). The influence of membrane lateral pressures on simple geometric models of protein conformational equilibrium. Chem. Phys. Lipids.

[19] Boheim G (1974). Statistical analysis of alamethicin channels in black lipid membranes. J. Membr. Biol..

[20] Zhou Y, Raphael RM (2007). Solution pH alters mechanical and electrical properties of phosphotidylcholine membranes: relation between interfacial electrostatics, intramembrane potential, and bending elasticity. Biophys. J..

[21] Keller SL, Mezrukov SM, Gruner SM, Tate MW, Vodyanoy I, Paresgian VA (1993). Probability of alamethicin conducting states varies with nonlamellar tendency of bilayer phospholipids. Biophys. J..

[22] Hanz SZ, Shu NS, Qian J, Christman N, Kranz P, An M, Grewer C, Qiang W (2016). Protonation-driven membrane insertion of a pH-low insertion peptide. Angew. Chem. Int. Ed..

[23] Alves DS, Westerfield JM, Shi X, Nguyen VP, Stefanski KM, Booth KR, Kim S, Morell-Falvey J, Wang BC, Abel SM, Smith AW, Barrera FN (2018). A novel pH-dependent membrane peptide that binds to epha2 and inhibits cell migration. eLife.

[24] Scott HL, Westerfield JM, Barrera FN (2017). Determination of the membrane translocation pK of the pH-low insertion peptide. Biophys. J..

[25] Scott HL, Nguyen VP, Alves DS, Davis FL, Booth KR, Bryner J, Barrera FN (2015). The Negative Charge of the Membrane has Opposite Effects on the Membrane Entry and Exit of pH-Low Insertion Peptide. Biochem..

[26] Anishkin A, Loukin SH, Teng J, Kung C (2014). Feeling the hidden mechanical forces in lipid bilayer is an original sense. Proc. Natl. Acad. Sci. U.S.A..

[27] Chiriac R, Luchian T (2007). pH modulation of transport properties of alamethicin oligomers inserted in Zwitterionic-based artificial membranes. Biophys. Chem..

[28] McClintic WT, Taylor GJ, Simpson ML, Collier CP (2020). Macromolecular crowding affects voltage-dependent alamethicin pore formation in lipid bilayer membranes. J. Phys. Chem. B.

[29] Maass W (1997). Networks of spiking neurons: the third generation of neural network models. Neural Netw..

[30] C.D. Schuman, T.E. Potok, S. Young, R. Patton, G.N. Perdue, G. Chakma, A. Wyer, G.S. Rose, “Neuromorphic Computing for Temporal Scientific Data Classification,” in *Proceedings of the Neuromorphic Computing Symposium* (Knoxville, TN, 2017), pp. 1–6

[31] M.S. Hasan, C.D. Schuman, J.S. Najem, R. Weiss, N.D. Skuda, A. Belianinov, C.P. Collier, S.A Sarles, G.S. Rose, “Biomimetic, Soft-Material Synapse for Neuromorphic Computing: From Device to Network,” in *Proceedings of the 2018 IEEE**13th Dallas Circuits and Systems Conference (DCAS) *(Dallas, 2018), pp. 1–6

[32] Najem JS, Taylor GJ, Armendarez N, Weiss RJ, Hasan MS, Rose GS, Schuman CD, Belianinov A, Sarles SA, Collier CP (2019). Assembly and characterization of biomolecular memristors consisting of ion channel-doped lipid membranes. J. Vis. Exp..

[33] Okazaki T, Sakoh M, Nagaoka Y, Asami K (2003). Ion channels of alamethicin dimer n-terminally linked by disulfide bond. Biophys. J..

