# Supplementary material for: Heterosynaptic plasticity in biomembrane memristors controlled by pH
Source: MRS Bull. 2022 Aug 29;48(1):13–21. doi: 10.1557/s43577-022-00344-z (PMC9988737; doi:10.1557/s43577-022-00344-z)
Supplement: Supplementary file 1 — Supplementary file1 (DOCX 3058 kb) [file 43577_2022_344_MOESM1_ESM.docx]

**Heterosynaptic plasticity in biomembrane memristors controlled by pH**

William T. McClintic,^1,†^ Haden L. Scott,^2,†^ Nick Moore,^3^ Mustafa Farahat,^4^ Mikayla Maxwell,^3^ Catherine D. Schuman,^5^ Dima Bolmatov,^6^ Francisco Barrera,^3^ John Katsaras,^2,6^ C. Patrick Collier ^1,7*^

^1^Bredesen Center for Interdisciplinary Research, University of Tennessee, Knoxville, Tennessee 37996; ^2^Large Scale Structures Group, Neutron Scattering Division, Oak Ridge National Laboratory, Oak Ridge, Tennessee 37831; ^3^Department of Biochemistry & Cellular and Molecular Biology, University of Tennessee, Knoxville, Tennessee 37996; ^4^Department of Chemical and Biomolecular Engineering, University of Tennessee, Knoxville, Tennessee 37996; ^5^Computer Science and Mathematics Division, Oak Ridge National Laboratory, Oak Ridge, Tennessee 37831; ^6^Shull Wollan Center, Oak Ridge National Laboratory, Oak Ridge, Tennessee 37831; ^7^Center for Nanophase Materials Sciences, Oak Ridge National Laboratory, Oak Ridge, Tennessee 37831

**Supporting Information**

**
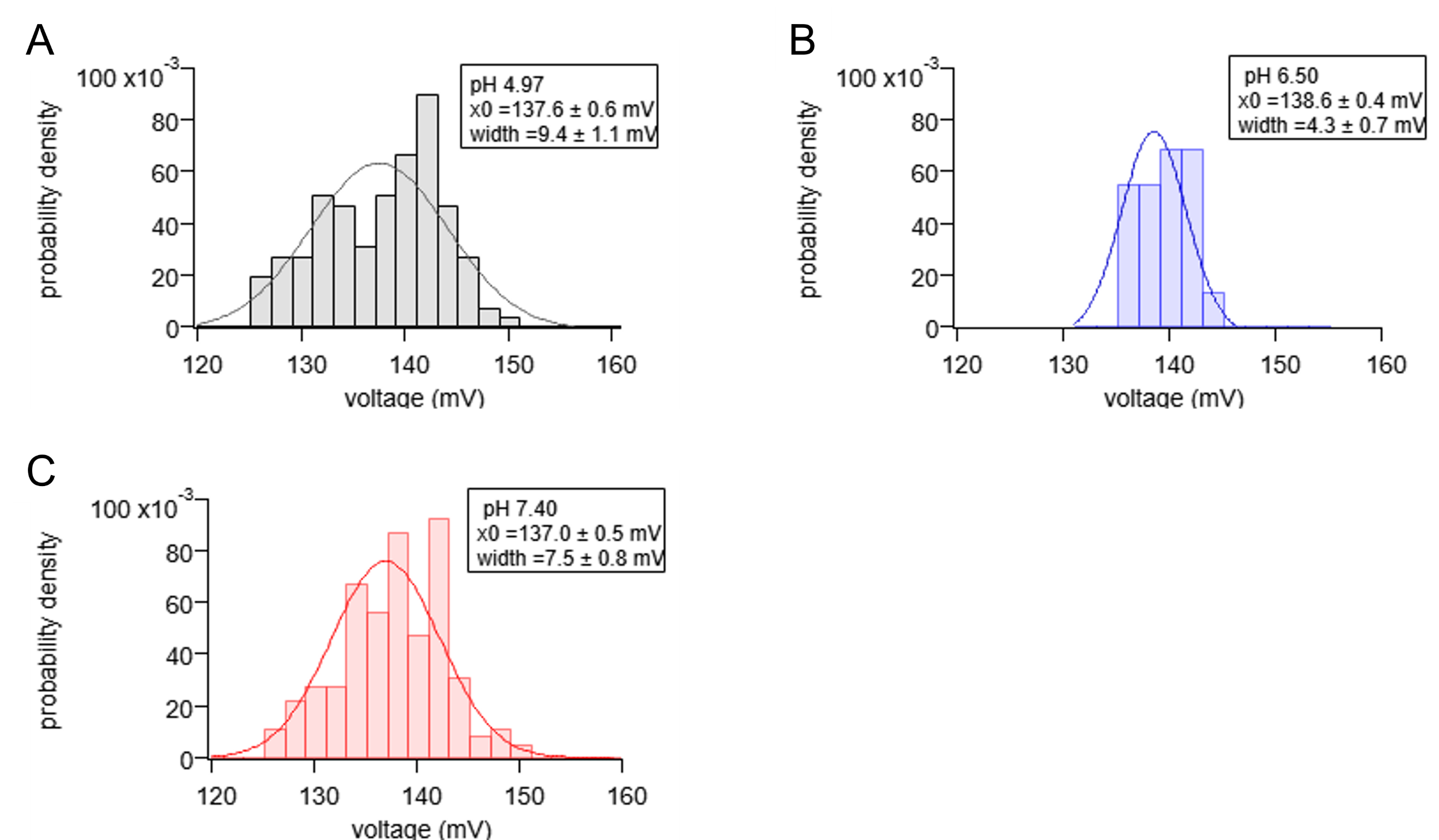
**

**Figure S1.** pH-dependent probability density distributions of *V** voltage thresholds for alamethicin pore formation in DPhPC lipid bilayers, fit to Gaussian functions. **A**. pH 4.97, **B.** pH 6.50, **C.** pH 7.40. The differences in their mean values (1-2 mV) were much smaller than the variances (width/2) of their distributions (4-9 mV), which indicates that noise levels were random and not correlated to changes in pH. At roughly the same scan rate (500 mV/s for pH 4.97 and 7.40, and 400 mV/s for pH 6.50), the hysteresis values (corresponding to the voltage differences when the alamethicin conductance exceeds the 8 μS/cm^2^ threshold in Figure 1C, then drops below it on the return leg back to 0V) for the three pH values were, on average, almost equivalent: ΔV=13 ± 1.8 mV.

**
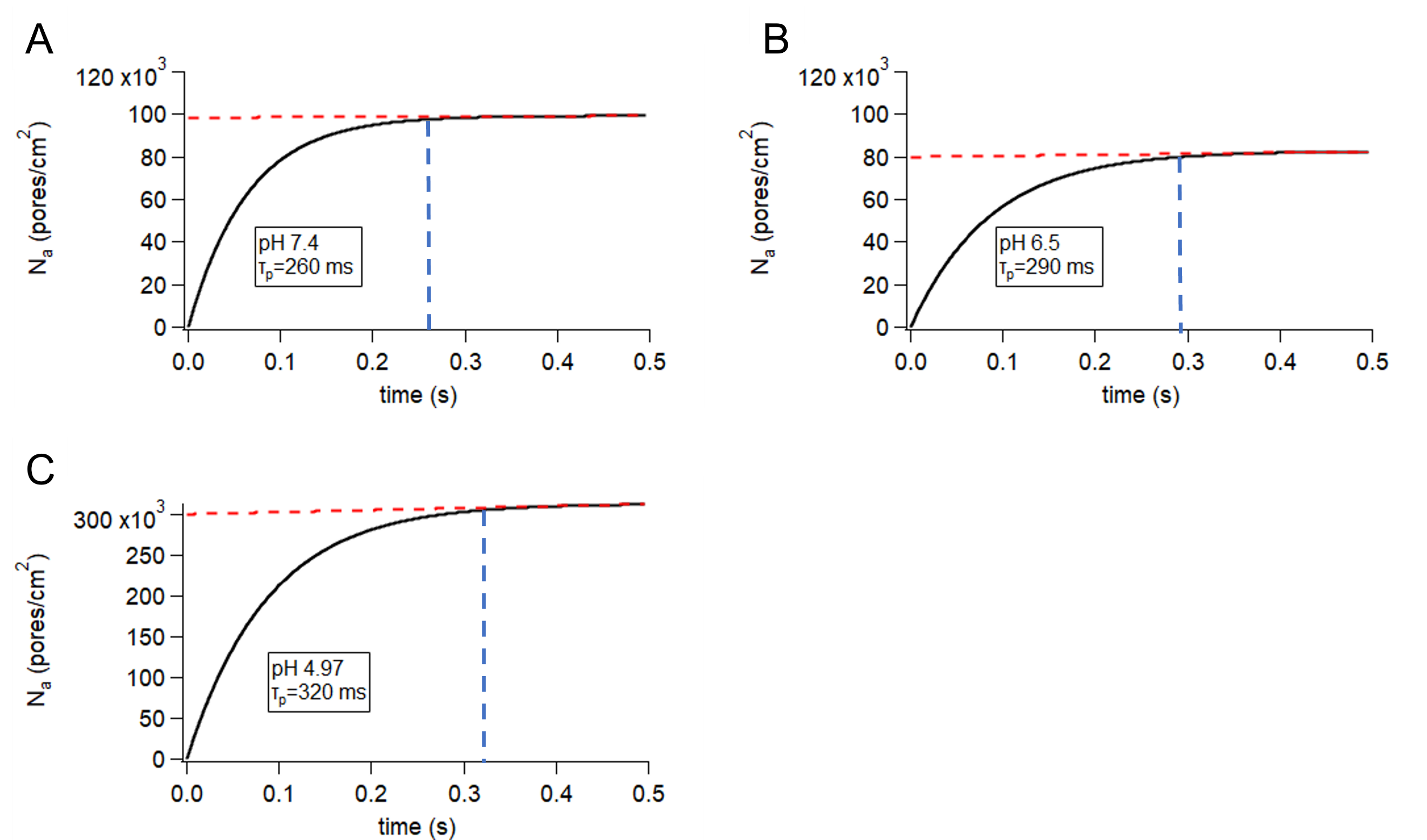
**

**Figure S2.** Determination of pH-dependent alamethicin potentiation times, τ_p_, which are the times needed for the number of open pores in the DPhPC membrane to reach steady-state values: *N_as_* = *n*/*m* (intersections of red and blue lines). This required that the electrowetting component to the ionic currents be removed by fitting them to Equation 9. **A**. pH 7.4, **B.** pH 6.50, **C.** pH 5.0.

**
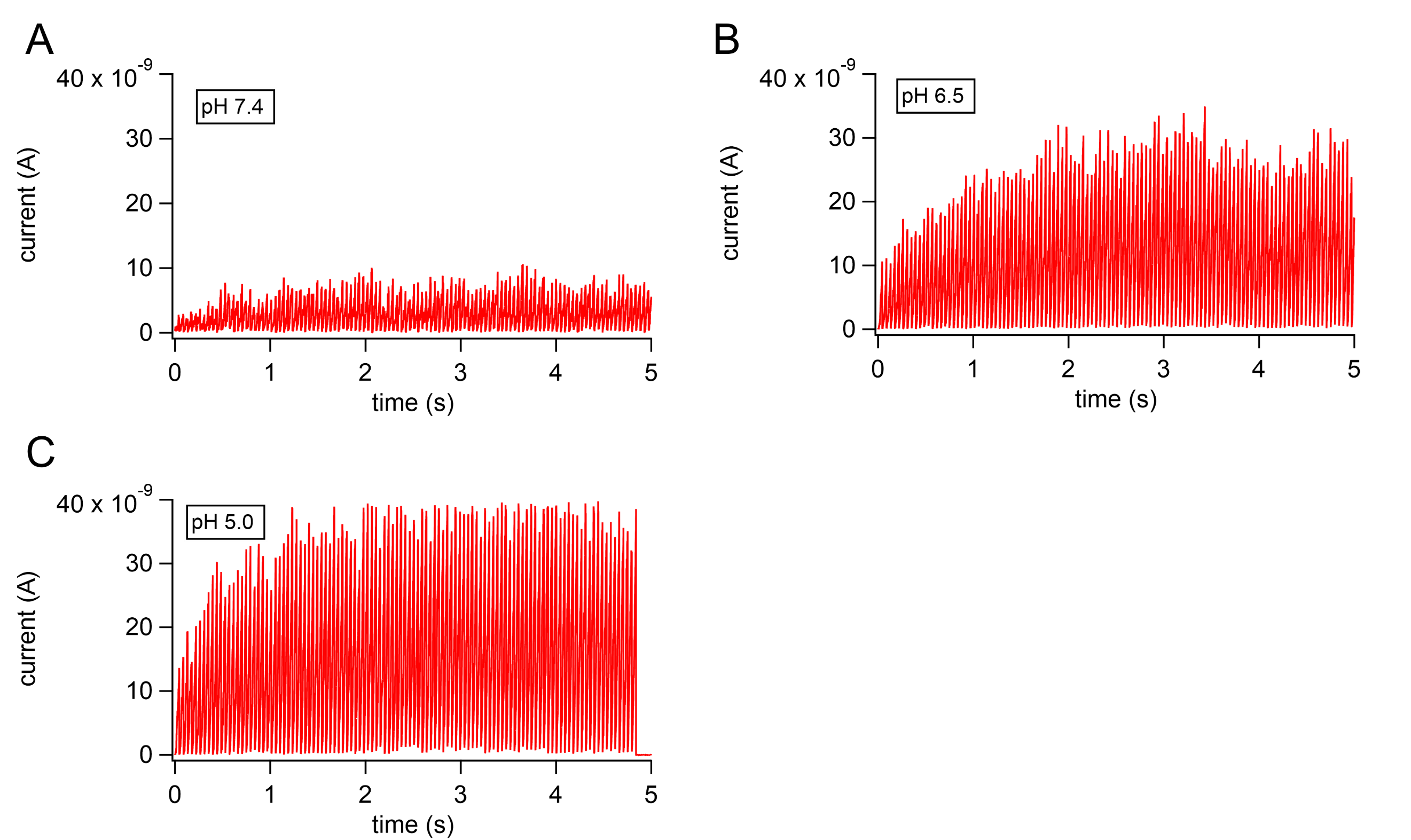
**

**Figure S3.** Changes in paired-pulse facilitation (PPF) versus pH from alamethicin-doped DPhPC memristors to a series of 145 mV, 50 ms pulses, separated by 10 ms off-times (5 seconds each, corresponding to 83 pulses). **A**. pH 7.4, **B.** pH 6.50, **C.** pH 5.0.

**
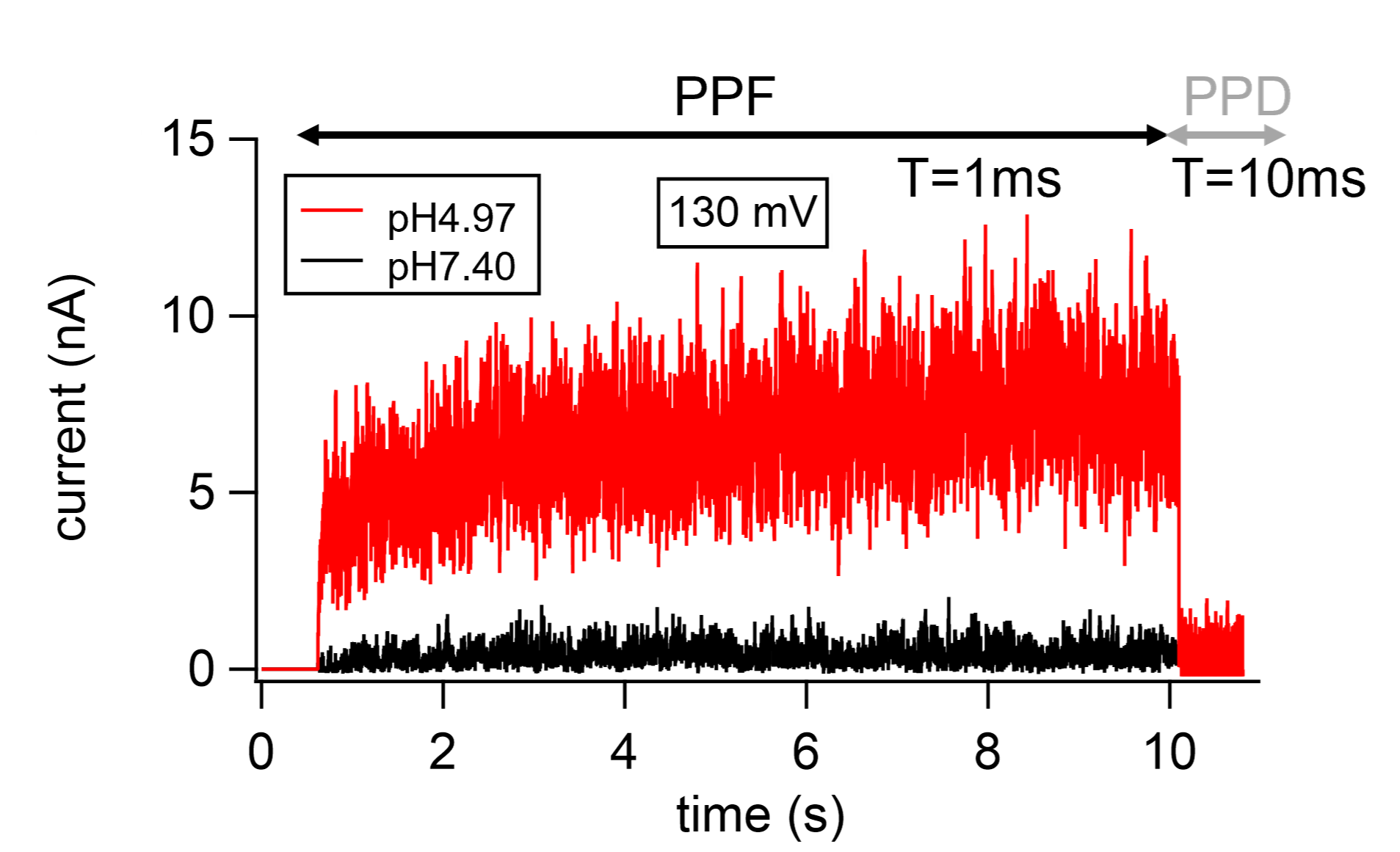
**

**Figure S4.** Changes in paired-pulse facilitation (PPF) and paired-pulse depression (PPD) versus pH from alamethicin-doped DPhPC memristors to a series of 130 mV, 20 ms pulses, separated by either 1 ms off-times (for PPF) or 10 ms off-times (for PPD). At the low bias voltage used here (130 mV), only the more acidic sample exhibited PPF and PPD. This contrasts with Figure S3, where PPF was observed for all pH values because the pulse voltage amplitudes were higher at 145 mV.
